# Antibody–Antibiotic Conjugates: Mechanisms, Clinical Progress, and Next‐Generation Strategies Against Multidrug‐Resistant Bacterial Infections

**DOI:** 10.1002/mbo3.70234

**Published:** 2026-02-18

**Authors:** Parvin Askari, Soudabeh Eshaghi, Leila Omidvar, Motahareh Mahi‐Birjand

**Affiliations:** ^1^ Infectious Diseases Research Center Birjand University of Medical Sciences Birjand Iran; ^2^ Clinical Research Development Unit (CRDU), Valiasr Hospital Birjand University of Medical Sciences Birjand Iran; ^3^ Department of Clinical Pharmacy, School of Pharmacy, Infectious Diseases Research Center Birjand University of Medical Sciences Birjand Iran

**Keywords:** antibiotic payload, antibody–antibiotic conjugates, bioconjugation, linker chemistry, multidrug‐resistant bacteria, targeted antimicrobial therapy

## Abstract

The rise of multidrug‐resistant (MDR) bacterial pathogens presents a critical challenge to global health, highlighting the need for innovative therapeutic strategies beyond conventional antibiotics. Antibody–antibiotic conjugates (AACs) combine the high specificity of monoclonal antibodies with the potent bactericidal activity of antibiotics, offering targeted delivery to extracellular and intracellular bacteria while minimizing off‐target toxicity. The present review provides a comprehensive analysis of AAC development, including key components, such as antigen selection, antibody engineering, linker chemistry, antibiotic payload optimization, and bioconjugation strategies. We summarize the mechanistic principles underlying AAC‐mediated bacterial clearance, emphasizing targeted payload release, fragment crystallizable region of the antibody (Fc)‐mediated immune engagement, and intracellular delivery. The temporal evolution of AACs is examined, highlighting milestones from early proof‐of‐concept studies to modern site‐specific, humanized constructs and emerging bispecific or dual‐payload designs. Furthermore, clinical development is discussed, focusing on pharmacokinetics, pharmacodynamics, safety, efficacy, and regulatory considerations, for example, intracellular infections and biofilm‐associated infectious agents. Current challenges, including antigen heterogeneity, immunogenicity, linker‐payload optimization, and manufacturing scalability, are critically analyzed, alongside strategies for next‐generation AACs. Collectively, AACs represent a transformative platform for precision‐targeted antimicrobial therapy, bridging gaps left by conventional antibiotics and offering a promising approach to combating MDR bacterial infections and associated clinical complications.

## Graphical abstract

Antibody–antibiotic conjugates enable targeted delivery of antibiotics to multidrug‐resistant bacteria by combining monoclonal antibody specificity with controlled intracellular drug release. This strategy improves efficacy against intracellular and biofilm‐associated infections while reducing systemic toxicity.

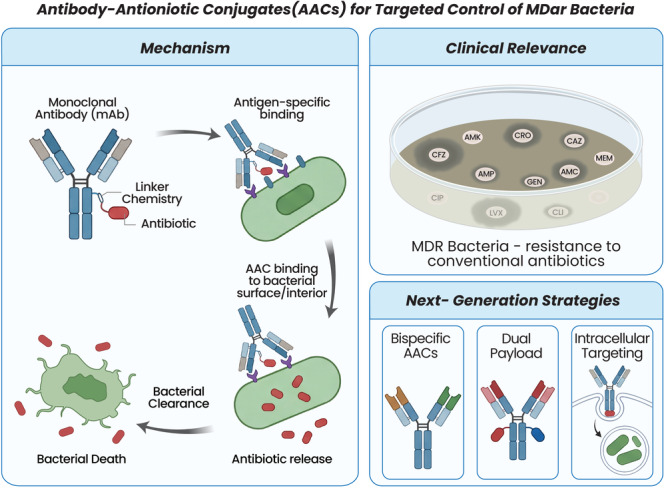

## 1 Introduction

Infectious diseases remain a leading global health threat, causing millions of deaths annually despite the widespread availability of antimicrobial agents (GBD 2019 Antimicrobial Resistance Collaborators 2022). The burden is particularly severe in low‐ and middle‐income countries, where bacterial infections contribute disproportionately to mortality and impose substantial socioeconomic costs (Boutzoukas and Doi 2025; Shafaie et al. 2025). Hospital‐acquired infections, community outbreaks, and chronic infectious diseases collectively strain healthcare systems (Institute of Medicine Forum on Microbial T 2010). This challenge is compounded by the rapid emergence of multidrug‐resistant (MDR) and extensively drug‐resistant bacterial strains, which undermine the effectiveness of existing antibiotics (Snobre et al. 2023; Beiki et al. 2022). The World Health Organization projects that antimicrobial resistance (AMR) could cause up to 10 million deaths per year by 2050 without novel interventions, highlighting the urgent need for innovative therapeutic strategies (Hanna et al. 2023).

The past decade has seen a marked decline in the discovery of new small‐molecule antibiotics due to scientific obstacles, economic limitations, and stringent regulatory requirements (Aslam et al. 2021). Traditional antibiotic development is becoming less and less effective, as candidate molecules often fail to overcome resistance mechanisms, such as efflux pumps, enzymatic degradation, target modification, and biofilm‐mediated tolerance (Aslam et al. 2021; Bell et al. 2014). Approved antibiotics frequently represent structural modifications of older classes, offering incremental increased benefits before resistance develops again (Roth et al. 2019). These limitations underscore the necessity of therapeutic approaches that circumvent conventional resistance mechanisms, enhance drug delivery precision, and minimize disruption to host microbiota (Dodds 2017; Askari et al. 2021).

Antibody–antibiotic conjugates (AACs) have emerged as a next‐generation antimicrobial platform, inspired by the success of antibody–drug conjugates (ADCs) in oncology (D'Amico et al. 2019; Khosravanian et al. 2024; Mer et al. 2024; Mirzaei et al. 2024). AACs combine the pathogen specificity of monoclonal antibodies (mAbs) with the bactericidal potency of antibiotics, enabling selective delivery of antibiotic payloads to bacterial cells or infected host compartments (Mariathasan and Tan 2017). This targeted approach enhances efficacy, systemic exposure, and toxicity (Mariathasan and Tan 2017; Darbandi et al. 2024). AACs are particularly promising against intracellular or biofilm‐associated pathogens, which are often inaccessible to conventional antibiotics (Darbandi et al. 2024).

Structurally, AACs consist of three key components: a pathogen‐targeting mAb, a linker, and a potent antibiotic payload for intracellular activity (Sun et al. 2009). The mAb directs the conjugate to the infection site, the linker enables controlled release, and the payload executes bactericidal activity locally (Govindan et al. 2016). Such a design facilitates selective accumulation of antibiotics at infection sites and, in some constructs, controlled release within immune cells harboring intracellular pathogens, promoting efficient bacterial clearance (Luo et al. 2025).

Preclinical evidence supports the potential of AACs. The THIOMAB‐based conjugate DSTA4637S, targeting *Staphylococcus aureus*, exemplifies this approach, in which a mAb binds bacterial teichoic acids and delivers a rifalogue payload (Peck et al. 2019). Following phagocytosis, intracellular conditions trigger linker cleavage, releasing the antibiotic inside macrophages (Luo et al. 2025; Peck et al. 2019). Animal studies demonstrated superior bacterial clearance and survival compared with free rifamycin or antibody alone, while early clinical studies showed a favorable safety profile and encouraging efficacy (Surur and Sun 2021; Deng et al. 2019). Similar strategies are being explored for Gram‐negative pathogens, though challenges such as outer membrane impermeability and antigen variability persist (Deng et al. 2019).

Despite progress, however, AAC development remains in its early stages. Critical considerations include antigen selection, antibody engineering, linker chemistry, payload optimization, and scalable bioconjugation (Deonarain and Yahioglu 2021). Ideal antigens must be abundant, conserved, and minimally cross‐reactive with host tissues (Pishesha et al. 2022). Linkers must balance systemic stability with controlled release, while payloads must retain bactericidal activity after conjugation (Sheyi et al. 2022). Manufacturing challenges, including conjugation stoichiometry, heterogeneity, and reproducibility, also require attention (Journeaux and Bernardes 2024). Furthermore, pharmacokinetic (PK) and pharmacodynamic (PD) modeling is complex, as AACs combine features of biologics and small molecules (Beck et al. 2017).

AACs offer strategic advantages beyond their targeted activity. By confining antibiotics to infection sites, they reduce off‐target effects on commensal microbiota and mitigate selective pressures that drive resistance (Mohammadzadeh et al. 2025). They also enable repurposing of otherwise toxic antibiotics and can be integrated into combination therapies with conventional antibiotics, host‐directed treatments, or novel approaches such as bacteriophages (de Nies et al. 2023).

Although literature on AACs has expanded to include mechanistic studies, linker and payload innovations, and early clinical investigations, a comprehensive synthesis mapping antigen and antibody strategies, linker chemistries, payload options, and translational outcomes remains limited (Tsuchikama et al. 2024). As AACs advance toward clinical application, integrative reviews are urgently needed to guide rational design, prioritize candidates, and identify development bottlenecks (Tao et al. 2025).

Accordingly, this systematic review provides a structured overview of AACs. We examine the global burden of infections, outline AAC components and design principles, explore mechanisms of action, review preclinical and clinical progress, and discuss challenges and future directions. By consolidating current knowledge and identifying gaps, this review aims to offer researchers, clinicians, and stakeholders a roadmap to advance AACs as safe, effective, and scalable solutions against AMR.

## 2 Key Requirements for the Design of AACs

**Figure 1:**
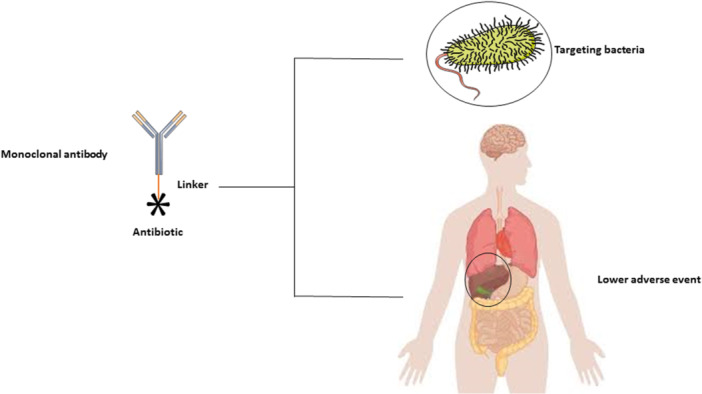
Characteristics of antibody–antibiotic conjugates (AACs). The schematic illustrates the basic structural components of an AAC. An antibiotic molecule is chemically conjugated to a monoclonal antibody via a cleavable or noncleavable linker, forming the conjugate. The linker plays a critical role in maintaining conjugate stability in circulation and enabling controlled antibiotic release at the target site. This design allows selective delivery of the antibiotic to bacterial cells recognized by the antibody, enhancing antibacterial efficacy while minimizing systemic toxicity.

AACs represent a promising class of targeted antimicrobial agents combining the specificity of mAbs with the potent bactericidal activity of antibiotics (Figure 1). The successful design of AACs requires careful optimization of several key components, including the antibody, linker, and antibiotic payload, as well as consideration of PK behavior and manufacturing feasibility (Colombo et al. 2024).

### 2.1 Antibody Component

The antibody serves as the targeting moiety that directs the conjugate toward bacterial surface antigens (Vacca et al. 2022). An effective antibody must recognize antigens abundantly expressed on the target bacteria and conserved across clinical isolates to ensure broad‐spectrum efficacy (H. Wang, Chen, et al. 2022). High affinity and specificity are essential for efficient bacterial binding and to minimize off‐target interactions with host tissues (Rostain et al. 2023). The antibody should also exhibit low immunogenic potential to reduce the risk of immune reactions during treatment (Carter and Quarmby 2024). Advances in antibody engineering, including the development of humanized and human mAbs, have significantly enhanced the therapeutic potential of AACs by improving target specificity and reducing immunogenicity (Kandari and Bhatnagar 2023).

### 2.2 Linker Chemistry

The linker plays a central role in connecting the antibody to the antibiotic payload and controlling the release of the active drug (Cavaco et al. 2022). It must remain stable in systemic circulation to prevent premature drug release, which can lead to systemic toxicity and reduced efficacy (Ezike et al. 2023). At the same time, it should be cleavable under specific conditions found at the infection site, such as acidic pH, enzymatic activity, or reducing environments, to ensure targeted antibiotic release (Devnarain et al. 2021). The linker and its byproducts should be biocompatible and nontoxic (Adepu and Ramakrishna 2021). Recent developments have introduced cleavable linkers incorporating disulfide bonds, hydrazone linkages, or enzyme‐sensitive peptide sequences that enable precise, environmentally triggered release of the antibiotic payload (Jadhav et al. 2025).

### 2.3 Antibiotic Payload

The antibiotic serves as the active component responsible for bacterial killing. It must be potent enough to eliminate the target pathogen and stable enough to withstand the conjugation and release processes without losing activity (Plotniece et al. 2023). Ideally, the antibiotic should have a broad antibacterial spectrum and a low tendency to promote resistance (Zacchino et al. 2017). Chemical compatibility with the linker is also essential to allow efficient conjugation without compromising antibiotic function (Yu et al. 2023). Ongoing research explores both traditional antibiotics and newly developed derivatives as payloads in AACs to overcome resistance mechanisms and expand therapeutic efficacy (Goldmacher and Kovtun 2011).

### 2.4 Drug‐to‐Antibody Ratio (DAR)

The DAR defines how many antibiotic molecules are attached to each antibody (Angiolini et al. 2025). This parameter directly affects the efficacy, stability, and safety of the conjugate (Pan et al. 2025). Higher DAR values generally increase antibacterial potency but may lead to greater toxicity and reduced stability, while lower DAR values can improve tolerability but may reduce antimicrobial activity (Karimi et al. 2023). Achieving an optimal DAR is crucial to balancing these effects and maximizing the therapeutic window (Irving and Gecse 2022). Modern site‐specific conjugation methods help ensure a consistent and controlled DAR across production batches (Hingorani 2024).

### 2.5 Pharmacokinetics and Pharmacodynamics

The PK and PD properties of AACs are determined by the combined influence of the antibody, linker, and antibiotic payload (Fan et al. 2024). The molecular size and structure of the conjugate affect its distribution, half‐life, and clearance in vivo (Lehar et al. 2015). The kinetics of antibiotic release, governed by linker stability and cleavage behavior, dictate the timing and duration of antimicrobial activity (Bellucci et al. 2024). Comprehensive pharmacokinetic–pharmacodynamic (PK/PD) modeling is therefore necessary to predict in vivo performance, design effective dosing regimens, and optimize therapeutic outcomes (L. Zhang et al. 2022).

### 2.6 Manufacturing and Scalability

Manufacturing AACs is complex, requiring precise bioconjugation techniques that ensure product quality, consistency, and scalability (Mu et al. 2022). Efficient conjugation processes are needed to achieve uniform DAR values and reduce batch‐to‐batch variability (M. Li, Zhao, et al. 2024). Purification steps must eliminate unconjugated components and maintain the homogeneity of the final product (Fernandez‐Cerezo et al. 2023). All manufacturing stages must comply with Good Manufacturing Practice (GMP) standards to ensure safety and regulatory approval. Recent advances in site‐specific conjugation and click chemistry have improved reproducibility and enabled scalable production of homogeneous AACs suitable for clinical use (Dudchak et al. 2024).

### 2.7 Antigen (Bacterial Antigens)

In AACs, the choice of the bacterial antigen is not a mere technicality but the very fulcrum upon which specificity, efficacy, and translational viability pivot (Blaskovich and Cooper 2025). Unlike cancer cell–targeted ADCs, AACs contend with the complex, variable, and often hostile environment of bacterial surfaces, including biofilms, polysaccharide capsules, and intracellular niches (David et al. 2019). As highlighted in Antibody–Antimicrobial Conjugates for Combating Antibiotic Resistance, antigen selection must balance three indispensable criteria: accessibility, conservation across strains, and functional importance to the pathogen (Yu et al. 2023).

From a mechanistic standpoint, an optimal antigen must be surface‐exposed under in vivo conditions, allowing the antibody moiety to bind without steric impediments or shielding by host biomolecules (Qian et al. 2023). In addition, expression should remain robust under varied growth states (e.g., biofilms, stationary phase, and osmotic stress), minimizing the risk of antigen downregulation or evasion (Schulze et al. 2021). The antigen ideally plays a critical physiological or virulence role—targeting such molecules imposes evolutionary constraints on the bacterium and makes escape less favorable (Smith et al. 2023).

In practice, AAC research has explored several classes of surface antigens. In Gram‐positive bacteria, wall teichoic acids (WTAs) and associated cell wall–anchored proteins are prominent targets (Zhydzetski et al. 2024). The canonical AAC developed by C. Zhou et al. (2016) (later known as DSTA4637A) conjugates a rifamycin derivative to a human immunoglobulin G1 (IgG1) mAb directed against WTA in *S. aureus*. This choice stems from WTA's relative conservation among *S. aureus* lineages and its accessibility at the cell surface. After binding, the AAC is internalized via phagocytosis, and lysosomal proteases (e.g., cathepsin B) cleave the valine–citrulline (Val‐Cit) linker, releasing the antibiotic within the phagolysosomal compartment (Qin et al. 2024).

In Gram‐negative species, antigen selection is more challenging due to the outer membrane barrier and extensive antigenic variability (Maher and Hassan 2023). Outer membrane proteins (OMPs), porins, and conserved core regions of lipopolysaccharide or lipooligosaccharide have been proposed as targets (Sakalauskienė and Radzevičienė 2025). However, the high antigenic diversity of *O*‐antigen polysaccharides often limits cross‐strain efficacy (Zahid et al. 2025). As Darbandi et al. (2024) emphasize in AACs: A Comprehensive Review, one useful strategy is to target more conserved core epitopes or hybrid designs combining carbohydrate and protein epitopes.

Capsular polysaccharides (CPSs), ubiquitous in many pathogenic bacteria (e.g., *Streptococcus pneumoniae* and *Klebsiella pneumoniae*), represent another antigenic class considered for AACs (Kajihara et al. 2021). Their inherent immunogenicity and surface exposure make them attractive; nonetheless, their great serotype diversity limits universal applicability (J. Zhou et al. 2022). To circumvent this, conjugate designs sometimes employ bispecific antibodies or mixtures of mAbs to broaden coverage (Sasso et al. 2023). Cavaco et al. (2021) underscore that antigen heterogeneity is a recurring challenge and must be addressed early in design.

Another aspect gaining attention is profiling antigen expression under realistic infection conditions. Many antigens characterized in vitro show different expression levels in vivo (e.g., within biofilms or intracellular environments), sometimes leading to reduced antibody binding (de Vor et al. 2022; Masters et al. 2022). The Comprehensive Review emphasizes this gap, recommending that antigen screening incorporate biofilm, stress, and in vivo–relevant models to avoid false positives (Loera‐Muro et al. 2021).

Case studies further illustrate both the promise and pitfalls. The WTA‐targeted AAC against *S*. *aureus* has shown efficacy in preclinical infection models by eliminating intracellular reservoirs inaccessible to free antibiotics (Wu et al. 2021). However, instances of antigen downregulation or epitope masking in certain host niches have been observed, underscoring a persistent risk of escape (Wong Fok Lung et al. 2022). Moreover, as The Use of AACs to Fight Bacterial Infections points out, antibody binding must often trigger or permit internalization; mere surface binding without uptake limits payload delivery (Cavaco et al. 2022).

Addressing these challenges, several mitigation strategies emerge from the literature. In this relation, targeting essential and conserved antigens makes escape less tolerable to the bacterium (B. Yang, Fang, et al. 2021). Employing bispecific or multispecific antibodies can buffer against antigenic variation (Amash et al. 2024). Engineering antibodies for broad paratope cross‐reactivity or increased affinity can compensate for epitope heterogeneity (Rojas 2022). Finally, combining AACs with matrix‐degrading enzymes or adjuvants may improve penetration into biofilms and overcome steric hindrance (Ahsan et al. 2024). In sum, the antigen choice is not merely the first design decision, it is a central determinant of AAC viability. The cumulative insights from recent evidence highlight that successful AACs require antigens that are accessible under in vivo infection conditions, conserved across clinical isolates, and functionally constrained. Meticulous antigen discovery and validation, preferably under clinically relevant environments, remain prerequisites for translating AACs from concept to robust therapeutics.

## 3 Structural Design and Bioconjugation Strategies for AACs

### 3.1 Antibody Component

The antibody serves as the defining determinant of specificity and functional behavior in AACs, governing both bacterial recognition and the pharmacological fate of the conjugate (Deka and Das 2025). Its design must harmonize high‐affinity antigen binding with optimal PK and immunological characteristics to ensure precise targeting and safe systemic administration (Guo and Nolan 2024). As emphasized in the relevant studies, the antibody is not merely a passive targeting scaffold but an active biological entity that dictates biodistribution, half‐life, immune interactions, and the intracellular trafficking pathway leading to antibiotic release (Yu et al. 2023; Kajihara et al. 2021).

Historically, the conceptual framework of AACs evolved from oncology‐derived ADCs. While both platforms rely on the principle of targeted payload delivery, their mechanistic contexts differ profoundly (Manzari et al. 2021). In oncology, antibodies typically bind to tumor‐associated antigens and internalize into malignant cells (Mendelsohn et al. 2025). Otherwise, in infectious disease, the bacterial targets may be extracellular, surface‐bound, or embedded within host cells (Bastounis et al. 2022). Consequently, the selection of antibody isotype, subclass, and structural format must be adapted to microbial pathophysiology rather than tumor biology (Song et al. 2023).

For effective bacterial targeting, antibodies used in AACs must recognize epitopes that are surface‐exposed, conserved, and accessible across clinical isolates (Ke et al. 2024). High‐affinity mAbs of the IgG1 subclass have predominated, owing to their balanced effector functions, stability, and manufacturability under GMP conditions (Covarrubias et al. 2022). The prototypical AAC, DSTA4637A, employs a humanized IgG1 directed against WTA in *S. aureus* (Wang‐Lin et al. 2018). This antibody not only provides antigen specificity but also facilitates opsonophagocytic uptake into macrophages, where protease‐sensitive linkers release the rifamycin payload (Deng et al. 2019; Wang‐Lin et al. 2018). The model exemplifies how the antibody's biological role, beyond simple recognition, can be exploited to extend antibiotic activity to intracellular bacterial reservoirs (Martín‐Galiano and McConnell 2019).

Recent reviews have highlighted antibody engineering as a central enabler of AAC development. Advances in humanization, phage display, and transgenic animal platforms have yielded fully human or humanized antibodies with reduced immunogenicity and enhanced serum stability (Cavaco et al. 2022; Q. Zhou 2017). Beyond conventional IgG formats, antibody fragments such as antigen‐binding fragment (Fab), single‐chain variable fragment (scFv), and nanobodies are being explored for improved tissue penetration and rapid clearance when prolonged exposure is undesirable (Y. Zhang et al. 2024). Bispecific and multispecific constructs that engage two distinct bacterial epitopes or combine bacterial and host receptors are also under investigation to enhance binding avidity and counteract antigenic heterogeneity (Darbandi et al. 2024).

An essential yet often underappreciated function of the antibody in AACs lies in its Fc‐mediated immune interactions. Fcγ‐receptor engagement can enhance opsonization and phagocytosis, promoting intracellular accumulation of the conjugate within macrophages and neutrophils; the very compartments that often harbor persistent pathogens (Edgar and Bournazos 2024). In this respect, AACs exploit not only antibody precision but also innate immune mechanisms (Tasin et al. 2022). Conversely, fragment crystallizable region (Fc) effector functions must be carefully modulated to avoid excessive inflammation or clearance; engineering silent or attenuated Fc variants can mitigate these risks in highly immunogenic infection sites (Bowman et al. 2024).

A major challenge in antibody selection arises from the diversity of bacterial antigens and infection niches (Grace et al. 2022). The antibody must retain binding capacity under conditions of fluctuating pH, ionic strength, and in the presence of host‐derived proteins that may mask bacterial surfaces (Tian et al. 2025). Moreover, in polymicrobial or biofilm‐associated infections, nonspecific adsorption and steric hindrance can reduce effective antibody engagement (Han and Poma 2022). Structural optimization of the Fab through affinity maturation or glycoengineering has been shown to improve resilience against such environmental variability (Pirkalkhoran et al. 2023).

Manufacturability and stability considerations also guide antibody design. Large‐scale AAC production requires antibodies with consistent glycosylation patterns and chemical robustness during linker conjugation (Matsuda and Mendelsohn 2021). Site‐specific conjugation strategies, such as engineered cysteine or enzymatic conjugation at defined residues, have markedly improved the homogeneity and DAR control of AACs (Khan et al. 2025). These developments, originally pioneered in oncology ADCs, have now been successfully translated to antimicrobial conjugates, enabling reproducible PK and safety profiles across batches (Bhushan and Misra 2024).

Despite these advances, several limitations persist. The high molecular weight of full‐length IgG molecules can restrict penetration into dense infection sites or biofilm matrices (Speziale and Pietrocola 2021). The relatively slow diffusion kinetics of antibodies may delay antibiotic release compared with small‐molecule prodrugs (Fralish et al. 2024). Moreover, antigenic drift, especially in Gram‐negative pathogens, threatens long‐term efficacy (López‐Siles et al. 2021). Addressing these issues requires integrating antibody discovery with genomic surveillance of clinical isolates and employing modular antibody scaffolds that can be rapidly re‐engineered in response to emerging resistance patterns (Ruan et al. 2024).

Collectively, the antibody component of AACs defines not only bacterial specificity but also the pharmacological, immunological, and translational trajectory of the entire conjugate. Future AAC success will hinge on the ability to design antibodies that combine high affinity and broad reactivity with optimal Fc engineering, manufacturability, and in vivo stability. As the latest reviews concur, the antibody is no longer a static delivery vehicle but a dynamic biological interface that harmonizes precision targeting with immune synergy as an essential evolution in the fight against MDR bacterial pathogens.

### 3.2 Linker Chemistry

The chemical linker in an AAC functions as the critical molecular bridge between the targeting antibody and the antimicrobial payload, and its properties are decisive for both therapeutic efficacy and safety (Lei et al. 2025). Unlike conventional antibiotics administered systemically, AACs rely on precise spatiotemporal release of the drug (Nazir et al. 2022), and the linker is the principal determinant of this controlled delivery. Recent reviews emphasize that linker design in AACs is not a trivial chemical consideration but a sophisticated engineering challenge that balances stability in circulation with efficient activation at the site of infection (J. Zhang et al. 2025; Yi et al. 2022).

A fundamental requirement of an effective linker is systemic stability. Premature cleavage of the antibiotic in circulation can result in off‐target toxicity, subtherapeutic concentrations at the infection site, and accelerated resistance development (MacNair et al. 2024). To address this, most AACs incorporate chemically stable linkers that resist serum proteases, hydrolysis, and reducing conditions encountered in plasma (L. Zhao et al. 2022). At the same time, the linker must be selectively cleavable under infection‐specific conditions. Current strategies exploit enzymatic activation by lysosomal or phagolysosomal proteases, pH‐sensitive hydrolysis in acidic microenvironments, or reduction of disulfide bonds within intracellular compartments (Yu et al. 2023; Sasso et al. 2023). For example, in the prototypical WTA‐targeted AAC against *S. aureus*, the rifamycin payload is released following cleavage of a Val‐Cit dipeptide linker by lysosomal cathepsin B after phagocytic uptake, ensuring that antimicrobial activity is concentrated within infected cells and intracellular bacterial reservoirs (Qin et al. 2024).

Another crucial aspect is linker biocompatibility and minimal intrinsic toxicity. Linkers must not generate reactive or immunogenic intermediates upon cleavage (Bray 2016). The use of nonimmunogenic peptide or hydrazone‐based linkers has been shown to reduce systemic adverse effects while preserving efficient drug release (Fu et al. 2022). Additionally, the chemical compatibility between the linker and both the antibody and the payload is essential; steric hindrance or inappropriate conjugation chemistry can compromise antigen binding, drug stability, or conjugate homogeneity (D. Su and Zhang 2021). This has motivated the adoption of site‐specific conjugation strategies, which enable controlled attachment at engineered cysteine residues or glycan sites, producing well‐defined DAR and minimizing heterogeneity (Walsh et al. 2021; Matsuda et al. 2025).

Recent advancements have explored environmentally responsive linkers, which exploit the unique chemical or enzymatic milieu of bacterial infection sites (X. Wang, Shan, et al. 2022). Acid‐sensitive linkers, disulfide bonds cleavable under reducing conditions, and enzyme‐activated peptide sequences are all under active investigation (X. Wang, Shan, et al. 2022). These approaches increase the selectivity of antibiotic release, concentrating the drug where pathogens reside while sparing uninfected tissues (Marzaman et al. 2023). As highlighted by Cavaco et al. (2022), such strategies are particularly advantageous for treating intracellular pathogens or biofilm‐associated infections, where traditional systemic antibiotics fail to reach effective concentrations.

Despite these advances, several challenges remain. Linkers must maintain stability during storage and large‐scale manufacturing, tolerate diverse antibody subclasses and payloads, and accommodate potential differences in bacterial microenvironments across patients (Foreman and Foreman 2025). Furthermore, the kinetics of cleavage must be optimized to balance rapid bacterial killing with sustained drug exposure, a task complicated by variable host‐cell uptake and intracellular trafficking patterns. Integrating computational modeling (Marei et al. 2022), structure–activity relationships, and in vitro infection models has increasingly informed rational linker design, allowing AACs to achieve therapeutic windows unattainable with free antibiotics (Breijyeh and Karaman 2023; Butler et al. 2024).

In summary, the linker is not merely a passive connector but a highly engineered component that dictates the spatial and temporal PDs of AACs. Advances in chemically stable, cleavable, and biocompatible linkers, combined with site‐specific conjugation techniques, have enabled precise control over antibiotic release and improved therapeutic indices. As highlighted across studies, rational linker design remains one of the most critical determinants of AAC success, directly influencing efficacy, safety, and translational potential.

### 3.3 Antibiotic Payload

The antibiotic payload is the functional centerpiece of an AAC, determining the bactericidal potency once the antibody has delivered the conjugate to the target site (Cavaco et al. 2022). Unlike conventional antibiotic therapy, where systemic exposure governs efficacy, AACs rely on the payload's ability to retain activity following conjugation, navigate intracellular or biofilm microenvironments, and overcome mechanisms of bacterial resistance (Stegemann and Trost 2023). The payload must not only be potent but also chemically compatible with the antibody and linker, preserving structural integrity throughout conjugation, circulation, and targeted release (D. Su and Zhang 2021).

Payload selection is guided by multiple interrelated factors. Potency is paramount, as the local concentration achievable at the infection site is limited by the stoichiometry of antibody binding (Lu et al. 2018). Highly potent antibiotics, such as rifamycin derivatives used in *S. aureus* AACs, can achieve bactericidal effects at low molar ratios, reducing the need for high DAR and minimizing potential off‐target toxicity (El‐Khoury et al. 2022). Moreover, the antibiotic must withstand the conjugation chemistry, systemic circulation, and enzymatic milieu of phagocytic compartments without degradation or loss of activity (Wright 2005).

Another critical consideration is the resistance profile of the antibiotic. Ideally, the payload should exhibit minimal cross‐resistance with existing therapies, maintaining efficacy against MDR pathogens (Gajic et al. 2025). The strategic use of antibiotics with novel mechanisms of action or derivatives specifically optimized for intracellular activity has emerged as a recurrent theme in the literature (AL‐Azzawi et al. 2024; Halawa et al. 2023). For instance, rifamycin derivatives employed in AACs are designed to evade efflux pumps and enzymatic inactivation, which frequently compromise conventional therapy (Peck et al. 2019). Similarly, vancomycin or daptomycin analogs are under investigation as AAC payloads for targeting Gram‐positive intracellular reservoirs (Darbandi et al. 2024).

Compatibility with the linker chemistry is also indispensable. Functional groups on the antibiotic must allow covalent attachment without altering its antimicrobial mechanism (Costa et al. 2011). Incompatibility can reduce payload release efficiency or inactivate the drug upon conjugation (Seidi et al. 2018). Recent literatures highlight advances in chemical engineering that enable selective derivatization of antibiotics while retaining pharmacological activity, such as site‐specific conjugation of rifamycin or glycopeptide derivatives to antibody‐linked peptide sequences (Hu et al. 2016; Drayton et al. 2020).

The choice of payload is further influenced by the infection niche. Intracellular pathogens or biofilm‐resident bacteria require antibiotics capable of penetrating host cells or extracellular polymeric substances (Martin et al. 2014). AACs targeting phagocytosed *S. aureus* employ lysosomally releasable payloads optimized for acidic, enzyme‐rich environments (Bray 2016). Similarly, payloads designed for Gram‐negative AACs must navigate outer membrane barriers or exploit host‐mediated delivery mechanisms to reach the bacterial cytoplasm (Bratkovič et al. 2024). These considerations underscore the integrated design principle in AACs: the antibody, linker, and payload must function as a coordinated unit rather than as isolated components (Cavaco et al. 2022).

In summary, the antibiotic payload is the linchpin of AAC function, requiring an intricate balance of potency, stability, resistance profile, chemical compatibility, and suitability for the target infection niche. As the recent literature underscores, successful AAC development depends on rational payload selection integrated with antibody and linker design, ensuring that the antimicrobial effect is both localized and maximized while minimizing systemic exposure and resistance development.

### 3.4 Bioconjugation

Bioconjugation is the central process in the development of AACs, serving as the molecular bridge that links the targeting antibody to the antimicrobial payload (Seixas et al. 2022). Unlike conventional antibiotics, which rely on systemic distribution to reach pathogens, AACs depend on precise, site‐specific delivery of the antibiotic, making the choice and method of conjugation a decisive factor for therapeutic success (Mariathasan and Tan 2017). The bioconjugation strategy directly influences DAR, structural homogeneity, PKs, antigen binding, payload release, and immunogenicity (Adhikari and Chen 2025).

Historically, the earliest conjugates employed random lysine or cysteine modification, capitalizing on the abundance of these residues in antibody structures (Haque et al. 2021). While technically straightforward, this approach produces heterogeneous mixtures with variable DARs, which can compromise antigen binding and induce unpredictable pharmacological behavior (Dong et al. 2024). Heterogeneity can lead to aggregation, altered clearance rates, or unintended immunogenicity, limiting translational potential (Lundahl et al. 2021).

To overcome these limitations, site‐specific conjugation strategies have emerged as the gold standard in modern AAC design. Site‐specific methods allow precise attachment of the antibiotic to defined residues or engineered motifs, producing uniform DARs and predictable PKs. Common strategies include:

1. *Engineered Cysteine Conjugation:* Antibodies are modified to introduce free thiol groups at predetermined positions, which react selectively with maleimide‐functionalized linkers. This allows controlled DAR, preserves the antigen‐binding Fab region, and minimizes structural perturbation (Szijj and Chudasama 2021).
2. *Enzymatic Conjugation:* Enzymes such as transglutaminase or sortase A mediate site‐specific attachment to engineered glutamine or glycine residues. This approach is highly selective, occurs under mild conditions, and maintains antibody folding and effector functions (Q. Zhou 2023).
3. *Bioorthogonal Click Chemistry:* Strain‐promoted azide‐alkyne cycloaddition or tetrazine–*trans*‐cyclooctene reactions allow rapid, chemoselective coupling under physiological conditions without interfering with antibody or payload structure (Debets et al. 2011). This method is particularly useful for sensitive antibiotics or complex linkers that are prone to hydrolysis or degradation (Debets et al. 2011; MacKenzie et al. 2014).

The choice of conjugation strategy is closely intertwined with linker chemistry and payload properties. For example, peptide‐based or enzyme‐cleavable linkers require precise orientation to ensure efficient intracellular release post‐phagocytosis, while sterically bulky or hydrophobic antibiotics may necessitate alternative conjugation sites to prevent antibody aggregation or loss of solubility (Lundahl et al. 2021). Studies have shown that site‐specific conjugation not only improves DAR uniformity but also preserves antigen binding, enhancing therapeutic index in preclinical infection models (Dong et al. 2024; Strop et al. 2015).

Beyond chemical precision, bioconjugation influences immunogenicity. Random conjugation may expose neoepitopes or alter Fc glycosylation patterns, potentially eliciting antidrug antibodies that reduce efficacy or accelerate clearance (Boune et al. 2020). By contrast, site‐specific conjugation preserves native antibody architecture, maintains Fc‐mediated effector functions when desired, and minimizes immunogenicity as an essential consideration for repeated dosing in chronic or recurrent infections (Delidakis et al. 2022).

Bioconjugation also dictates PK behavior. Homogeneous AACs with controlled DAR exhibit more predictable serum half‐lives, tissue distribution, and clearance rates compared with heterogeneous conjugates (Holz et al. 2023). This is critical because intracellular delivery kinetics and controlled payload release are central to AAC efficacy, particularly against pathogens residing within phagocytes or biofilms (Cavaco et al. 2022).

Recent innovations aim to integrate adaptive and modular conjugation platforms, allowing rapid reengineering of antibodies or payloads in response to emerging bacterial resistance (Baker et al. 2018). For example, engineered conjugation sites can accommodate different classes of antibiotics or multiple payloads, creating bispecific or multiantibiotic AACs capable of targeting heterogeneous bacterial populations or polymicrobial infections (Cavaco et al. 2022; Mariathasan and Tan 2017).

Finally, scalability and manufacturability are central to clinical translation. Modern site‐specific conjugation techniques are compatible with GMP standards, allowing reproducible production of AACs with consistent DAR, purity, and activity across batches (M. Li, Zhao, et al. 2024). This reproducibility is critical not only for regulatory approval but also for ensuring predictable safety and efficacy in human trials (Yadav et al. 2024; Pusztai et al. 2013).

In conclusion, bioconjugation in AACs is a multifaceted, highly engineered process that integrates antibody structure, linker chemistry, and payload characteristics. The transition from heterogeneous, random conjugation to precise, site‐specific methodologies has dramatically improved therapeutic potential by enhancing stability, PKs, and immunogenic safety. As current research demonstrates, future success in AAC development will depend on further innovations in modular, site‐specific, and bioorthogonal conjugation strategies that allow rapid adaptation to diverse pathogens and resistance mechanisms.

## 4 Mechanism of Action of AACs

AACs operate through a multilayered mechanism that combines the precision targeting of antibodies with the bactericidal potency of antibiotics, representing a transformative strategy against MDR bacterial infections (C. Zhou et al. 2016). Unlike traditional antibiotics, which diffuse systemically and often fail to reach intracellular or biofilm‐embedded pathogens, AACs provide spatially and temporally controlled delivery, maximizing antimicrobial efficacy while minimizing systemic toxicity (Deng et al. 2019).

The mechanism begins with highly selective antigen recognition. The antibody component binds to bacterial surface antigens, which can include WTAs, CPSs, OMPs, or conserved virulence factors (Kurokawa et al. 2016). Selection of the target antigen is critical: It must be broadly expressed across clinical isolates, highly accessible, and ideally internalizable through host phagocytic pathways (Pan et al. 2014). High‐affinity antibodies ensure rapid accumulation at infection sites, reducing off‐target interactions and enhancing the therapeutic index (Darbandi et al. 2024; Mariathasan and Tan 2017).

Following antigen engagement, internalization and intracellular trafficking become central, particularly for pathogens residing within host phagocytes (Mantegazza et al. 2013). AACs targeting *S. aureus* demonstrate this principle: antibody binding promotes opsonization and phagocytosis, directing the conjugate into macrophage or neutrophil compartments (Lehar et al. 2015). Within the acidic, enzyme‐rich environment of phagolysosomes, cleavable linkers, for example, typically dipeptide or peptide‐hydrazone linkers undergo proteolytic or chemical cleavage, releasing the antibiotic payload directly in proximity to the intracellular bacteria (Peck et al. 2019). This targeted release circumvents traditional PK limitations, enabling intracellular antibiotic concentrations that free drugs often fail to achieve.

The antibiotic payload then exerts its bactericidal effect, leveraging canonical mechanisms such as inhibition of RNA polymerase, protein synthesis, or cell wall assembly (Al‐Tohamy and Grove 2025). The precise localization of the antibiotic within intracellular compartments or biofilms allows effective bacterial killing at lower systemic doses (Pals et al. 2024). Importantly, AACs can overcome multiple resistance mechanisms: the conjugated payload bypasses efflux pumps, avoids extracellular enzymatic inactivation, and retains activity against persistent phenotypes (Pham et al. 2019; Homer et al. 2025).

Fc‐mediated immune functions complement the direct antimicrobial activity. Antibody Fc domains facilitate opsonization, activate complement, and promote phagocytosis, enhancing bacterial clearance (Koenderman 2019). Intact Fc domains augment immune‐mediated killing, while attenuated Fc variants reduce inflammation at sensitive sites (T. H. Kang and Jung 2019). This dual mechanism, direct antibiotic killing and immune‐mediated clearance, represents a distinctive advantage over conventional antibiotic therapy (Ankomah and Levin 2014; Gjini and Brito 2016).

AACs also show enhanced activity against biofilm‐associated bacteria, which are typically refractory to free antibiotics due to reduced penetration and altered metabolic states (Tvilum et al. 2023). The antibody directs the conjugate to biofilm surfaces, while enzymatically or pH‐sensitive linkers release antibiotics that penetrate the biofilm matrix and act on embedded bacterial populations (Yu et al. 2023). This targeted approach achieves therapeutic concentrations at sites where conventional systemic antibiotics fail, offering potential for eradication of chronic or device‐associated infections (Yu et al. 2023; Tvilum et al. 2023).

Recent innovations have expanded AAC mechanisms through multispecific targeting and payload diversification. Bispecific or multispecific antibodies can simultaneously recognize multiple bacterial antigens or combine pathogen recognition with host‐cell targeting, increasing binding avidity and overcoming antigenic heterogeneity (Kontermann 2012; J. J. Kang et al. 2023). In parallel, payload diversification strategies, including dual antibiotics or novel antibiotic derivatives, enable sequential or synergistic mechanisms of killing while reducing the likelihood of resistance emergence (Pantaleo et al. 2022; Abebe and Birhanu 2023).

Payload release dynamics are another critical determinant of AAC efficacy. The timing, location, and kinetics of antibiotic liberation depend on linker chemistry, intracellular trafficking, and host–pathogen interactions (Munguia and Nizet 2017). Cleavage must be rapid enough to exert bactericidal activity yet controlled to minimize premature systemic release (Munguia and Nizet 2017; Chiang et al. 2018). Studies demonstrate that optimized can maintain sustained intracellular antibiotic levels sufficient to eradicate persisters without increasing systemic toxicity (Drayton et al. 2020; Homer et al. 2025; Peukert et al. 2023).

Furthermore, AACs can be designed to target extracellular bacteria, intracellular reservoirs, and biofilm niches concurrently, offering a comprehensive antimicrobial strategy. This versatility is particularly important for pathogens, such as *S. aureus*, *K. pneumoniae*, and *Pseudomonas aeruginosa*, which exhibit both intracellular persistence and biofilm formation (Faria et al. 2023). The ability to integrate antigen specificity, antibody effector function, cleavable linker chemistry, and highly potent payloads underpins the AAC platform's success in preclinical models (Cavaco et al. 2022; Chan et al. 2025; Bebbington and Yarranton 2008).

In summary, the mechanism of AACs is a sophisticated interplay of antigen recognition, targeted internalization, controlled intracellular or extracellular antibiotic release, and synergistic immune‐mediated bacterial clearance. This multilayered strategy allows AACs to overcome traditional pharmacological barriers, achieve localized therapeutic concentrations, and address MDR and biofilm‐associated infections that are otherwise difficult to treat. The mechanistic versatility of AACs, combined with the rational design of antibody, linker, and payload positions, positions this platform as a transformative approach in antimicrobial therapy.

## 5 Temporal Milestones of AACs

The evolution of AACs reflects a multidecade effort to merge the specificity of mAbs with the potent bactericidal activity of antibiotics, offering a targeted solution to MDR infections (Qin et al. 2024). AAC development can be understood as a sequence of overlapping scientific and technological milestones that trace the progression from conceptual frameworks to translational and preclinical achievements, culminating in modern clinical‐stage AAC candidates (Cavaco et al. 2022; Ye and Chen 2022). Table 1 presents an overview of ACCs, detailing their clinical or preclinical applications, molecular targets, and comparative advantages and disadvantages.

**Table 1:** List of antibacterial antibody–antibiotic conjugates, their used disease, targets, advantages, and disadvantages.

AAC name/alias	Pathogen(s) studied	Antibody target/payload (linker)	Main advantages	Main disadvantages/limits	Development phase/studies	Reference
DSTA4637A/DSTA4637S (RG7861) (THIOMAB TAC)	*Staphylococcus aureus* incl. MRSA (intracellular reservoirs; bacteremia models)	mAb against *S. aureus* surface (wall teichoic acid) conjugated to a rifamycin‐class payload (dmDNA31/rifalogue) via a protease‐cleavable linker (THIOMAB site‐specific conjugation).	Targets bacteria for Fc‐mediated uptake into phagocytes and releases rifamycin locally—increases intracellular killing, reduces off‐target systemic exposure.	Complex manufacture; potential rifamycin resistance; possible immunogenicity and linker stability concerns.	Phase 1/1b complicated *S*. *aureus* bacteremia (NCT02596399, NCT03162250)	Peck et al. (2019)
Anti‐*Pseudomonas* AAC (mAb 26F8–G2637)	*Pseudomonas aeruginosa* (preclinical models; intracellular in phagocytes)	mAb (26F8) binding LPS *O*‐antigen + conjugated G2637 (arylomycin analog targeting type I signal peptidase LepB) via cathepsin‐cleavable linker.	Potent intracellular killing; enables reuse of antibiotics with modest extracellular potency.	Antigen heterogeneity (LPS variability); Gram‐negative penetration challenges; preclinical only.	Preclinical (in vitro)	Kajihara et al. (2021)
KRM‐1657 payload AAC (multiple antibody backbones)	*S. aureus* (MRSA)—intracellular models *and* in vivo MRSA models	Antibodies directed at *S*. *aureus* conjugated to KRM‐1657 (rifamycin derivative) via cleavable linkers.	Elimination of intracellular MRSA in vitro/in vivo; potent MICs versus MRSA.	Preclinical; potential off‐target release and resistance risk; manufacturing optimization needed.	Preclinical (in vitro)	Fan et al. (2024)
Other experimental AACs (polymyxin/peptide payloads, OM‐disrupting conjugates)	Gram‐negatives (*P*. *aeruginosa*, *Klebsiella*, *Acinetobacter baumannii*)	Antibodies delivering antimicrobial peptides, polymyxin derivatives, or OM‐disrupting moieties.	Expand the antibiotic toolbox, reduce systemic toxicity by targeted delivery.	In vitro/animal only; antigen diversity; toxicity concerns with polymyxin payloads.	Preclinical (in vitro)	Krivić et al. (2022)

Abbreviations: AAC, antibody–antibiotic conjugate; LepB, Leader peptidase I; LPS, lipopolysaccharide; mAb, monoclonal antibody; MICs, minimum inhibitory concentrations; MRSA, methicillin‐resistant *Staphylococcus aureus*; OM, outer membrane.

### 5.1 Early Conceptual Foundations (Pre‐2000s)

The conceptual basis of AACs was heavily influenced by ADCs in oncology (Riccardi et al. 2023). Early work demonstrated that antibodies could selectively deliver cytotoxic molecules to diseased cells, minimizing systemic toxicity (Tsuchikama 2019). Translating this concept to infectious diseases, researchers hypothesized that antibiotics could similarly be targeted to bacterial pathogens through high‐affinity antibodies (Cavaco et al. 2022). Initial studies were largely exploratory, focusing on the feasibility of antigen‐specific binding and payload delivery, without sophisticated linker chemistry or optimized conjugation methods (Sasso et al. 2023; Alradwan et al. 2025). These foundational insights established that antigen specificity, antibody effector function, and payload potency were critical determinants of targeted antimicrobial efficacy (Jiang et al. 2011; Odoom et al. 2025).

### 5.2 First‐Generation AACs (2000–2010)

During this period, AACs transitioned from concept to proof‐of‐principle constructs. Early designs relied on random lysine‐ or cysteine‐based conjugation to attach conventional antibiotics to murine antibodies (Matsuda et al. 2025). While these constructs demonstrated selective targeting to bacterial surfaces, they suffered from heterogeneous DAR, unpredictable PKs, and immunogenicity due to nonhuman antibody frameworks (Qian et al. 2023; Kennedy et al. 2017). Nevertheless, preclinical studies established key principles: antibodies could direct antibiotics to extracellular bacteria, facilitate opsonophagocytosis, and enhance killing of intracellular pathogens in vitro and in animal models (Jin et al. 2022; Shim 2020). These studies also highlighted limitations, including premature drug release, low intracellular accumulation, and inconsistent therapeutic outcomes (C. L. Li, Ma, et al. 2024; Elshiaty et al. 2021).

### 5.3 Second‐Generation AACs (2010–2017)

Technological breakthroughs during this era addressed the limitations of first‐generation constructs. Site‐specific conjugation strategies emerged, employing engineered cysteine residues, enzymatic tags, or bioorthogonal chemistries to produce homogeneous AACs with defined DARs and improved PKs (Lim 2020; Agarwal and Bertozzi 2015). Humanized and fully human antibodies replaced murine frameworks, minimizing immunogenicity and enabling repeated dosing (Harris and Cohen 2024). Linker design became increasingly sophisticated, incorporating cleavable sequences responsive to intracellular proteases or acidic pH, allowing precise payload release within phagocytes or biofilm microenvironments (Giese et al. 2021). Preclinical models during this period demonstrated enhanced clearance of *S. aureus* and other MDR Gram‐positive pathogens, establishing proof‐of‐concept for intracellular targeting and biofilm penetration (Yu et al. 2023; C. Zhou et al. 2019; H. Cai et al. 2020).

### 5.4 Translational and Preclinical Milestones (2017–2023)

The period between 2017 and 2023 marked a rapid acceleration in AAC development, bridging preclinical innovation with translational potential. Fully human mAbs were paired with highly potent antibiotic payloads, including rifamycin derivatives and glycopeptide analogs optimized for intracellular activity (Yu et al. 2023; Dartois et al. 2025). Site‐specific conjugation and advanced linker chemistries allowed precise control over DAR, drug release kinetics, and systemic stability, addressing heterogeneity and off‐target toxicity (Bellucci et al. 2024; Z. Zhao et al. 2020).

Notably, AACs demonstrated efficacy against both extracellular bacteria and intracellular reservoirs within macrophages, highlighting their unique mechanism compared with free antibiotics (Baz et al. 2024). Several studies confirmed the ability of AACs to penetrate biofilms and achieve therapeutically relevant intracellular antibiotic concentrations, overcoming persistent infections that are resistant to conventional therapy (Darbandi et al. 2024; Z. Cai et al. 2025; Q. Xu et al. 2021). Preclinical safety and efficacy studies provided the foundation for first‐in‐human evaluations, validating PK models, DAR optimization, and linker stability as critical design parameters (Pretto and FitzGerald 2021; Q. Huang et al. 2024).

### 5.5 Emerging Innovations (2023–Present)

Recent advancements emphasize modular AAC platforms, enabling rapid adaptation to emerging pathogens, novel antibiotics, or multitarget strategies (Mishra et al. 2025). Bispecific antibodies allow simultaneous recognition of multiple bacterial antigens, increasing binding avidity and overcoming antigenic heterogeneity (Labrijn et al. 2019). Dual or sequential antibiotic payloads offer complementary mechanisms of action, reducing the risk of resistance development (Sullivan et al. 2020; Baym et al. 2016).

Integration of computational modeling, in vitro infection systems, and animal models has refined design parameters, such as optimal DAR, linker cleavage kinetics, and intracellular release profiles. Concurrently, scalable GMP‐compatible bioconjugation methods now permit reproducible production of homogeneous AACs with predictable pharmacology and minimal immunogenicity, a critical milestone for clinical translation (Lombardi et al. 2025; González‐Outeiriño et al. 2005).

### 5.6 Key Takeaways and Future Perspectives

The temporal milestones of AACs underscore a trajectory from conceptual frameworks to sophisticated, clinically actionable therapeutics. Each phase of development from early experimental constructs to modern bispecific and dual‐payload platforms reflects iterative improvements in antibody engineering, linker chemistry, payload selection, and bioconjugation (McCombs and Owen 2015). The accumulated knowledge provides a blueprint for addressing current and future challenges, including MDR pathogens, intracellular persistence, biofilm‐associated infections, and rapid adaptation to emerging bacterial threats (Zafer et al. 2024; Goshisht 2025).

In conclusion, the historical evolution of AACs highlights the synergistic interplay of biological, chemical, and engineering innovations. Understanding these temporal milestones provides critical context for designing next‐generation AACs with optimized efficacy, safety, and translational potential, positioning them as a transformative tool in the fight against antibiotic‐resistant infections.

## 6 Clinical Development of AACs

The translation of AACs from preclinical proof‐of‐concept studies to clinical applications represents a critical milestone in antimicrobial therapeutics. Leveraging the specificity of mAbs and the bactericidal potency of antibiotics, AACs offer a promising solution to MDR infections, intracellular pathogens, and biofilm‐associated bacterial communities (Cavaco et al. 2022). The clinical development pathway of AACs illustrates the integration of advanced antibody engineering, sophisticated linker chemistry, optimized payload selection, and rigorous preclinical validation to achieve safe and effective therapies (Carter and Rajpal 2022; Liu et al. 2025).

### 6.1 Preclinical Evaluation and Translational Foundations

Prior to clinical testing, AAC candidates undergo extensive preclinical evaluation, focusing on PKs, PDs, safety, and efficacy (Brennan et al. 2015). Preclinical studies in murine and nonhuman primate models have demonstrated that AACs can achieve targeted bacterial killing in both extracellular and intracellular compartments, particularly in macrophages infected with *S. aureus* (Rasheed et al. 2021 *)*. These studies also establish the importance of controlled DAR, site‐specific conjugation, and linker stability in determining therapeutic index (K. Xu et al. 2013; Hui et al. 2022).

Toxicology studies confirm that site‐specific AACs exhibit reduced systemic toxicity compared with free antibiotics, owing to the targeted delivery mechanism and minimized off‐target exposure (Nazir et al. 2022; X. Yang, Ye, et al. 2021). Moreover, the immune‐modulatory role of the antibody Fc domain enhances opsonophagocytosis and complements direct payload activity, further supporting translational potential (Kim et al. 2012; Proctor et al. 2021).

### 6.2 Early‐Phase Clinical Trials

Initial clinical development has focused on Phase I studies to evaluate safety, tolerability, PKs, and immunogenicity (Postel‐Vinay et al. 2016). Humanized or fully human mAbs have been critical in mitigating antidrug antibody responses, a key concern for repeated dosing in bacterial infections (Darbandi et al. 2024; Alradwan et al. 2025). Early‐phase trials have also explored dosing regimens, DAR optimization, and linker‐payload combinations to achieve sufficient intracellular and extracellular concentrations without systemic toxicity (Drago et al. 2021; López de Sá et al. 2023; Conilh et al. 2023).

These studies have confirmed that AACs are generally well tolerated, with adverse events largely related to infusion reactions or transient immune activation rather than antibiotic toxicity. PK analyses indicate prolonged half‐life relative to free antibiotics, attributable to the antibody carrier, and favorable tissue distribution, including accumulation at infection sites (Darbandi et al. 2024; Cavaco et al. 2022).

### 6.3 Target Pathogens and Clinical Indications

AACs in clinical development have primarily targeted MDR Gram‐positive pathogens, particularly *S. aureus*, including methicillin‐resistant strains (Qin et al. 2024). Emerging constructs also address Gram‐negative pathogens, biofilm‐associated infections, and intracellular reservoirs, expanding potential indications to prosthetic‐device infections, endocarditis, and chronic osteomyelitis (Fan et al. 2024). The selection of target antigens such as WTAs, CPSs, and OMP ensures high specificity, broad clinical applicability, and reduced off‐target effects (Cavaco et al. 2022; Fan et al. 2024).

### 6.4 Advanced Clinical Studies and Proof‐of‐Concept

Phase II and early‐Phase III studies have begun to explore efficacy endpoints, including bacterial clearance, reduction of biofilm burden, and infection resolution in patients with MDR infections. These trials often incorporate PD markers, such as intracellular bacterial killing, opsonophagocytic activity, and immunological correlates, to better understand the mechanistic contributions of AACs beyond conventional antibiotics (Mariathasan and Tan 2017; Darbandi et al. 2024; Deng et al. 2019).

Notably, preclinical evidence suggests that AACs maintain efficacy against antibiotic‐resistant strains that are refractory to conventional therapy, offering a strategic advantage in hospital‐acquired infections and difficult‐to‐treat cases (Deng et al. 2019; Staben et al. 2016). Early clinical data support these findings, demonstrating meaningful reductions in bacterial load and favorable safety profiles (Baah et al. 2021; Gheibi Hayat and Sahebkar 2020). Moreover, the modularity of AACs allows rapid adaptation to emerging pathogens or resistance mechanisms, an essential feature in the era of escalating AMR (Yu et al. 2023; Kajihara et al. 2021).

### 6.5 Regulatory and Manufacturing Considerations

Successful clinical translation of AACs requires adherence to GMP and regulatory standards, ensuring consistent DAR, purity, and homogeneity across production batches (Khongorzul et al. 2020; Matsuda et al. 2019). Site‐specific conjugation methods and bioorthogonal chemistries facilitate reproducible manufacturing at scale, addressing one of the historical barriers to clinical translation (Negash et al. 2019). Regulatory agencies increasingly recognize AACs as distinct from traditional antibiotics, emphasizing both antimicrobial activity and antibody safety, including immunogenicity and Fc‐mediated effects (Mariathasan and Tan 2017; Behrens and Liu 2014).

## 7 Current Challenges and Next‐Generation AACs

AACs represent a transformative approach to combating MDR bacterial infections, yet several scientific, technical, and translational challenges must be addressed to realize their full clinical potential. Lessons from preclinical studies, early‐phase clinical trials, and advances in ADCs inform the ongoing evolution of next‐generation AACs (Darbandi et al. 2024).

### 7.1 Antigen Selection and Bacterial Heterogeneity

A primary challenge lies in identifying appropriate bacterial antigens that are highly expressed, accessible, and conserved across clinical isolates. Heterogeneity in antigen expression among strains, serotypes, or growth phases can reduce targeting efficiency and therapeutic outcomes (Kailayangiri et al. 2020). For example, variation in WTAs, CPSs, or OMPs may compromise AAC binding, particularly in biofilm‐associated or intracellular reservoirs (Arciola et al. 2015). Addressing this challenge requires careful antigen screening and may involve bispecific or multispecific antibodies capable of recognizing multiple epitopes to broaden coverage and overcome antigenic variability.

### 7.2 Antibody Engineering and Immunogenicity

Although humanized and fully human antibodies have reduced immunogenicity, repeated or high‐dose AAC administration may still elicit antidrug antibody responses, potentially limiting efficacy (Howard et al. 2025). Fc‐mediated immune effects are double‐edged; while enhancing opsonophagocytosis and complement activation, they may also trigger inflammation or off‐target immune responses in sensitive tissues (Morgan and Harris 2015). Next‐generation AACs explore Fc‐engineered antibodies to fine‐tune immune engagement, balancing bacterial clearance with host safety.

### 7.3 Linker and Payload Optimization

The linker chemistry remains a critical determinant of AAC performance. Premature cleavage can release antibiotics systemically, causing toxicity, whereas overly stable linkers may fail to release the payload at the site of infection, compromising efficacy (F. Y. Su et al. 2018). Advances in environmentally responsive linkers activated by intracellular enzymes, acidic pH, or bacterial‐specific conditions have improved payload release precision, yet further optimization is needed for diverse infection niches (Yu et al. 2023; Cheng and Wuest 2019).

Payload selection also presents challenges. Antibiotics must retain activity post‐conjugation and exhibit minimal propensity for resistance development (Devanga Ragupathi et al. 2019). The choice of payload impacts DAR, solubility, and conjugation efficiency (Buecheler et al. 2018). Emerging strategies include dual or sequential antibiotic payloads, novel derivatives resistant to efflux or enzymatic inactivation, and combinations tailored for biofilm or intracellular targeting (Elshobary et al. 2025; Nadar et al. 2022; Niño‐Vega et al. 2025).

### 7.4 Pharmacokinetics, Pharmacodynamics, and Dosage

Predicting AAC distribution, half‐life, and effective intracellular concentrations is complex due to their hybrid biological–chemical nature (Volak et al. 2023). DAR, antibody size, linker stability, and target engagement collectively influence PKs and PDs (Gao et al. 2024). Optimizing dosing regimens requires sophisticated PK/PD modeling to achieve sufficient tissue penetration and intracellular accumulation while minimizing systemic exposure and toxicity (Dong et al. 2024; Gao et al. 2024).

### 7.5 Manufacturing, Scalability, and Regulatory Hurdles

AACs involve intricate bioconjugation processes that must produce homogeneous, reproducible products with controlled DAR, high purity, and stability. Site‐specific conjugation, click chemistry, and enzymatic approaches have improved consistency, yet scaling up for clinical‐grade production remains challenging (Schumacher et al. 2014). Regulatory evaluation requires integration of traditional antibiotic metrics with antibody safety assessments, including immunogenicity, Fc‐mediated effects, and pharmacology, which complicates approval pathways (Sadowsky et al. 2017).

### 7.6 Biofilm and Intracellular Targeting

AACs must efficiently penetrate biofilms and intracellular compartments to achieve therapeutic outcomes. Biofilm matrices impede diffusion, and intracellular pathogens often reside in low‐pH, enzyme‐rich phagosomes (Kirtane et al. 2021). Next‐generation designs focus on cleavable linkers activated in microenvironments, antibody engineering to enhance cellular uptake, and payloads optimized for intracellular stability. Additionally, targeting biofilm‐specific antigens or using bispecific antibodies may enhance delivery to these challenging infection niches (Qin et al. 2024; Le et al. 2021).

### 7.7 Next‐Generation AAC Strategies

Emerging next‐generation AAC strategies are being developed to overcome current limitations and broaden therapeutic potential. One promising direction involves the design of bispecific and multispecific AACs, which can simultaneously target multiple bacterial antigens, thereby improving binding avidity and ensuring more comprehensive coverage against heterogeneous bacterial populations (S. Huang et al. 2020). Another innovative approach is the incorporation of dual or sequential antibiotic payloads, which can reduce the likelihood of resistance development while promoting synergistic bactericidal mechanisms (Morales‐Durán et al. 2024). Furthermore, the use of environmentally responsive linkers allows controlled drug release triggered by specific physiological cues such as pH, bacterial enzymes, or metabolic byproducts, resulting in enhanced site‐specific delivery (Raza et al. 2019).

Recent advances have also introduced modular and rapidly adaptable AAC platforms that facilitate the swift development of novel constructs against emerging and MDR Gram‐negative pathogens (Cavaco et al. 2022). In addition, integration with immune modulation represents another frontier, where Fc‐engineered antibodies are optimized to strengthen immune effector functions, particularly opsonophagocytic activity, while minimizing excessive inflammatory responses (Darbandi et al. 2024; Cavaco et al. 2022). Overall, these innovations underscore the dynamic evolution of AAC design toward more targeted, adaptive, and effective antibacterial therapies.

## 8 Discussion

Recent progress in AACs has shifted the field from conceptual promise to a mechanistically grounded and translationally credible therapeutic platform (Sasso et al. 2023). Early AAC studies primarily demonstrated feasibility; However, contemporary research has clarified the determinants of efficacy, particularly antigen selection, intracellular trafficking, linker‐controlled payload release, and PK/PD integration (Qian et al. 2023; Cavaco et al. 2022). Among these advances, the development of site‐specific conjugation technologies represents a major milestone, enabling homogeneous AACs with defined DARs, improved stability, and predictable in vivo behavior (Hingorani 2024; Dong et al. 2024). These innovations directly address limitations of first‐generation constructs, where heterogeneity and premature payload release undermined translational potential.

Mechanistically, AAC research has advanced beyond surface targeting to elucidate host‐cell–mediated delivery paradigms, especially for intracellular pathogens (Varma et al. 2020). The prototypical *S. aureus*–directed AACs have demonstrated that antibody‐mediated opsonophagocytosis followed by lysosomal linker cleavage enables localized antibiotic release within infected phagocytes (Ke et al. 2024). This intracellular targeting mechanism represents a fundamental conceptual advance over conventional antibiotics, which often fail to reach sufficient intracellular concentrations (Epand et al. 2016). Importantly, AACs have been shown to overcome resistance mechanisms such as efflux, enzymatic degradation, and metabolic dormancy by bypassing extracellular barriers and delivering high local payload concentrations (Alidriss et al. 2025).

**Figure 2:**
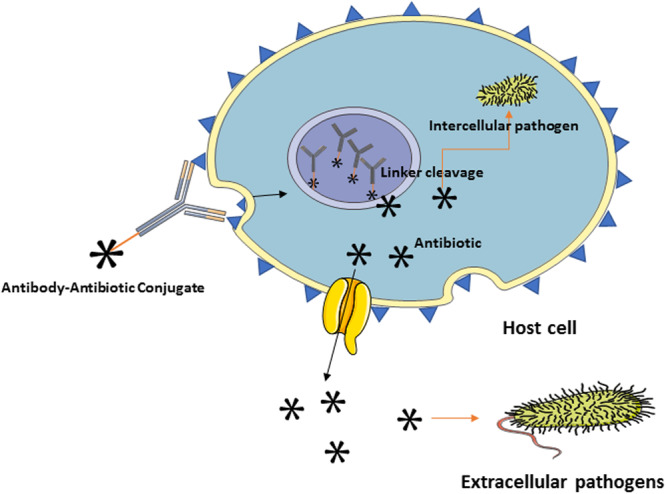
Schematic representation of antibody–antibiotic conjugate structure and mechanism of action.

Significant progress has also been made in linker and payload optimization, informed by lessons from oncology‐derived ADCs (Fujii and Matsuda 2023). Protease‐cleavable and environmentally responsive linkers now allow precise spatial and temporal control of antibiotic release, minimizing systemic exposure while maximizing antibacterial activity (Xue et al. 2021). Parallel efforts in payload engineering have enabled the repurposing of antibiotic classes, such as rifamycins and glycopeptides, whose clinical utility was previously limited by toxicity or PK constraints (Barman et al. 2019; Nguyen et al. 2019). These advances underscore that AAC efficacy is an emergent property of coordinated antibody, linker, and payload design rather than any single component (Figure 2).

From a translational perspective, AACs have progressed into early clinical evaluation, marking a critical inflection point for the field. Phase I studies of anti‐*S*. *aureus* AACs have demonstrated acceptable safety profiles, prolonged systemic half‐life relative to free antibiotics, and PK behavior consistent with controlled payload release (Lehar et al. 2015; C. Zhou et al. 2016). Although clinical efficacy data remain limited, these trials validate the feasibility of AACs as a therapeutic class and highlight the importance of rigorous PK/PD modeling to guide dose selection and clinical indication (Zhu et al. 2025).

Despite this progress, substantial challenges remain. Antigen heterogeneity, particularly among Gram‐negative pathogens, continues to limit broad‐spectrum applicability (Darbandi et al. 2024). In addition, differences between in vitro antigen expression and in vivo infection niches complicate target validation (Yu et al. 2023). Current research increasingly emphasizes bispecific antibodies, conserved antigen targeting, and modular AAC platforms to address these limitations (Mariathasan and Tan 2017). Manufacturing scalability and regulatory classification also remain unresolved, as AACs occupy a hybrid space between biologics and small‐molecule antibiotics (Foreman and Foreman 2025).

Collectively, recent research advances position AACs not as incremental refinements of existing antibiotics but as a mechanistically distinct antimicrobial modality. The convergence of antibody engineering, linker chemistry, and translational pharmacology has transformed AACs into a rationally designed platform capable of addressing intracellular persistence, biofilm tolerance, and multidrug resistance. Future progress will depend on continued integration of mechanistic insight with clinical development, rather than descriptive expansion, to fully realize the therapeutic potential of AACs.

## 9 Conclusion

AACs represent a promising frontier in antimicrobial therapeutics, merging the precision of mAbs with the potent bactericidal activity of antibiotics to combat MDR infections. By integrating targeted antigen recognition, controlled payload release, and Fc‐mediated immune activation, AACs address many of the shortcomings of conventional antibiotics, including inadequate intracellular penetration, biofilm tolerance, and diverse resistance mechanisms.

Over time, AAC development has evolved from conceptual prototypes to highly refined, site‐specific, and humanized constructs incorporating optimized linkers, DAR, and conjugation chemistries. Recent innovations such as bispecific targeting, dual or sequential antibiotic payloads, and environmentally responsive linkers have expanded their therapeutic versatility and resistance‐mitigating potential. Encouragingly, early clinical studies have demonstrated favorable safety, predictable PKs, and preliminary efficacy against MDR Gram‐positive and intracellular pathogens. Despite this progress, several challenges remain, including antigenic heterogeneity, immunogenicity, and manufacturing scalability. Addressing these will require integration of computational modeling, high‐throughput antigen discovery, and modular design strategies to accelerate AAC optimization and deployment.

In essence, AACs herald a new era in precision antimicrobial therapy, bridging the divide between biologics and small‐molecule antibiotics. With continued innovation, these conjugates are poised to transform the treatment landscape and make a meaningful contribution to the global effort against antibiotic resistance.

## Author Contributions

**Parvin Askari:** conceptualization, literature search, data curation, writing – original draft. **Soudabeh Eshagh:** literature search, data interpretation, writing – review and editing. **Leila Omidvar:** critical revision of the manuscript, provision of clinical perspective, supervision. **Motahareh Mahi‐Birjand:** conceptualization, methodology, supervision, project administration, writing – review and editing, final approval of the version to be published.

## Funding

The authors received no specific funding for this work.

## Ethics Statement

During the preparation of this work, the authors used ChatGPT only to correct grammar and spelling. Following the use of this tool, the authors thoroughly reviewed and edited the content as needed and take full responsibility for the content of this publication.

## Conflicts of Interest

The authors declare no conflicts of interest.
